# Disparities in Health Care Utilization by Smoking Status – NHANES 1999–2004

**DOI:** 10.3390/ijerph6031095

**Published:** 2009-03-13

**Authors:** Jennifer W. Kahende, Bishwa Adhikari, Emmanuel Maurice, Valerie Rock, Ann Malarcher

**Affiliations:** Centers for Disease Control and Prevention, National Center for Chronic Disease Prevention and Health Promotion, Office on Smoking and Health, 4770 Buford Highway, NE., MS-K50, Atlanta, Georgia 30341, USA; E-Mails: Badhikari@cdc.gov (B.A.); Emaurice@cdc.gov (E.M.); Vrock@cdc.gov (V.R.); Amalarcher@cdc.gov (A.M.)

**Keywords:** Smoking, tobacco use, health care, cessation, utilization

## Abstract

The objective of this study was to assess disparities in health care utilization, by smoking status, among adults in the United States. We used 1999–2004 National Health and Nutrition Examination Survey (NHANES) data from 15,332 adults. Multivariate logistic regressions were used to examine the relationship between smoking status (current, former, and never smoker), with health care utilization. After controlling for demographic characteristics, current smokers and former smokers who quit either <2 years or ≥10 years prior to the survey were more likely to have had inpatient admission in the past year than never smokers. Current smokers did not differ from never smokers on whether they had an outpatient visit in the past year. They were, however, more likely than never smokers to have ≥4 outpatient visits. Smokers who quit either <2 years ago or ≥10 years ago were more likely to have had an outpatient visit than never smokers. Former smokers were more likely than never smokers to have ≥4 outpatient visits regardless of when they quit. Our results show that cigarette smoking is associated with higher health care utilization for current and former smokers than for never smokers. Frequent hospitalization and outpatient visits translate into higher medical costs. Therefore, more efforts are needed to promote interventions that discourage smoking initiation and encourage cessation.

## 1. Introduction

Tobacco use is the leading preventable cause of morbidity and mortality in the United States [1]. An estimated 20.8% of American adults (≥18 years of age) currently smoke cigarettes [2] and annually, cigarette smoking results in approximately 443,000 deaths [3]. The annual economic cost of smoking in the United States is estimated at $193 billion ($96 billion in direct health care expenditures and $97 billion in lost productivity) [3,4]. Both current smokers and former smokers experience higher health care utilization and incur higher medical expenditures than never smokers [5–9]. Data from randomized, controlled clinical trials indicate that differences in outpatient and inpatient health care utilization by smoking status exist [10]. They observed that former smokers increased their average outpatient visits by approximately two visits during the first year of cessation, while outpatient health care use was relatively unchanged among continuing smokers [10]. Within four years of cessation, however, both inpatient and outpatient health care utilization were significantly lower among former smokers than those who continued smoking. Another study found that higher health care utilization and costs appear to begin prior to cessation due to a major health event that may have prompted cessation [11]. Increases in health care utilization during the first year of cessation may be due to follow-up care for an acute health event or recurrence of the acute health event that led to the decision to quit smoking [11].

Socio-demographic characteristics are important determinants of health care utilization in the United States and need to be considered when assessing the relationship between smoking and health care utilization. For example, women are more likely to utilize health care than men, as are elderly persons with lower levels of income/less education, and those with health insurance [12]. Most other studies on health care utilization and smoking have looked at selected populations such as members of health care management organizations (HMOs) [6–11,13].

Important to note are recent changes in patterns of smoking. For example, cigarette smoking among adults has decreased dramatically during the last 40 years [2] and widespread implementation of effective tobacco control programs and policies has been found to be effective at reducing initiation, increasing smoking cessation, and reducing exposure to secondhand smoke [14,15]. In addition, the number of effective cessation therapies now available has increased over time along with insurance coverage for effective cessation treatments [16,17]. At the same time more tobacco products are being introduced into the marketplace than ever before, and smokers are more likely to use multiple tobacco products which will have an additional impact on their health [4,15,18–22]. Patterns of who is quitting and when they quit may be changing, and these changes in the populations of current and former smokers may be affecting their health care utilization.

The purpose of the current study was to assess disparities in health care utilization by smoking status among a national sample of adults. It is important to look at this question in a population-based study because samples from HMOs tend to be among healthier people and of higher economic status than the general population. It is also important to use recent national data because of the multiple changes in smoking status.

## 2. Methods

To estimate health care utilization by smoking status of U.S. adults, we used 1999–2004 National Health and Nutrition Examination Survey (NHANES) data. NHANES is a nationally-representative survey conducted by the Centers for Disease Control and Prevention (CDC) to assess the health and nutritional status of adults and children in the United States through interviews and direct physical examinations. The surveys contain information on current and past smoking behaviors, health care utilization, and demographic characteristics of the study population. The sample size in this study consisted of 15,332 adults (≥18 years of age) and the data for the analyses was weighted.

Self-reported inpatient utilization was measured by whether respondents were hospitalized in the past year and by the number of times respondents were hospitalized in the past year. Self-reported outpatient utilization was measured by whether respondents had an outpatient visit with a medical provider in the past year and by the total number of visits respondents had in the past year. For the analysis, the number of inpatient admissions and outpatient visits were each grouped into two levels. For inpatient utilization, we looked at those who indicated that they had been hospitalized and also who indicated that they had been hospitalized ≥2 times in the past year. Outpatient visits were also categorized as those who had an outpatient visit in the past year and those who indicated they had ≥4 outpatient visits in the past year. Respondents were asked whether they had smoked at least 100 cigarettes in their entire life to classify their smoking status. Those who answered “yes” were asked whether they now smoke cigarettes every day, some days, or not at all. For our analysis, current smokers were those who had smoked at least 100 cigarettes during their lifetime and, at the time of the interview, reported smoking either every day or some days. Former smokers were those who reported smoking at least 100 cigarettes during their lifetime but currently did not smoke. We grouped former smokers into four categories based on the length of time since they quit smoking (<2 yrs, 2–4 yrs, 5–9 yrs, and ≥10 yrs). Never smokers were those who reported never having smoked 100 cigarettes during their lifetime. Multivariate logistic regression models were used to assess the relationship between smoking status (current, former, and never smoker) and health care utilization controlling for the following respondent demographic characteristics: gender, race/ethnicity, age, education, poverty level (based on year of survey), and health insurance coverage using SUDAAN 9.0.1 (Research Triangle Institute, Cary, North Carolina). Our referent groups for smoking status and demographic characteristics included never smoker, male, Non-Hispanic white, those 18–24 years old, those with more than a high school education, those living at or above poverty level, and those with no health insurance coverage. We used the Wald statistic to determine whether the odds ratios were statistically significant (p<0.05).

## 3. Results

Among 15,332 respondents who completed the surveys, 24.9% were current smokers, 50.2% were never smokers and 25.0% were former smokers. Among all respondents, 3.3% reported quitting less than 2 years prior to the survey, 2.6% quit 2–4 years prior, 3.2% quit 5–9 years prior, and 15.9% quit ≥10 years prior to the survey.

Table 1 shows the distribution of demographic characteristics of current, former, and never smokers. Current (54.1%) and former (50.8%–57.1%) smokers included a higher proportion of men than never smokers (40.8%). The highest proportion of respondents were Non-Hispanic whites. Current smokers were younger than never smokers and former smokers who quit smoking for ≥2 years. Over one-half of never and former smokers had more than a high school education; this proportion was higher than that of current smokers (39.1%). Current smokers were more likely to have incomes below the poverty level (18.9%) and to have no health insurance coverage (30.1%) than former smokers (6.5%–13.2% and 6.3%–20.3%) or never smokers (11.7% and 15.2%).

Former smokers who quit smoking less than 2 years prior to the survey had a higher proportion of hospitalization in the past year (19.6%) compared with current smokers (10.7%), never smokers (10.0%), former smokers who quit 5–9 years ago (11.1%), and former smokers who quit ≥10 years ago (12.9%). Among those who had been hospitalized in the past year, current smokers (22.9%) and never smokers (21.1%) had been hospitalized ≥2 times. Regardless of smoking status, the majority of respondents had at least one outpatient visit in the past year. However, 23.3% of current smokers had no outpatient visits in the past year, compared with 8.3%–17.9% of those in the other groups. Among those who had an outpatient visit in the past year, former smokers were more likely to have ≥4 outpatient visits (47.8%–52.0%) than current smokers (42.0%) or never smokers (42.1%).

### 3.1. Multivariate Analysis

Table 2 shows the adjusted odds ratios (OR) along with 95% confidence intervals (CIs) for the variables in the model. After controlling for smoking status, gender, race/ethnicity, age, education, poverty level and health insurance, current smokers (OR=1.20, CI, 1.06–1.37) and former smokers who had quit for <2 years (OR=2.49, CI, 1.86–3.34) or ≥10 years (OR=1.22, CI, 1.02–1.46) were significantly more likely to have been hospitalized in the past year than never smokers; smoking status, however, was not associated with the number of times hospitalized in the past year. Those who quit either <2 years ago (OR=1.75, CI, 1.15–2.65) or ≥10 years ago (OR=1.75, CI, 1.42–2.14) were more likely to have had an outpatient visit than never smokers. Current smokers (OR=1.18, CI, 1.06–1.33) and former smokers were more likely to have had ≥4 outpatient visits compared to those who had never smoked (p<0.05).

Women, survey respondents ≥65 years of age, persons with ≤ high school education, those with incomes below the poverty level, and persons with health insurance were more likely to have had a hospitalization in the past year than their respective referent groups. Among respondents who were hospitalized, the only sociodemographic characteristics associated with an increased number of hospital stays were having an income below the poverty level (OR=1.50, CI, 1.05–2.13). Women (OR=2.90, CI, 2.56–3.30), persons aged ≥45 years of age (OR=1.26, CI, 1.02–1.56 for persons 45–64 years of age and OR=2.77, CI 2.16–3.55 for persons ≥65 years of age), and persons with health insurance (OR=3.15, CI, 2.67–3.73) were more likely to have had an outpatient visit in the past year than referent group. Mexican Americans (OR=0.72, CI, 0.62–0.85) and persons with less than a high school education (OR=0.75, CI, 0.62–0.92) were less likely to have had an outpatient visit in the past year. Women, persons aged ≥45 years of age, persons living below the poverty level, and persons with health insurance were also related to increased frequency of visits; the relation between having less than a high school education and frequency of outpatient visits, however, was reversed - they were more likely than those with more than a high school education to have more frequent visits.

## 4. Discussion

Adjusted multivariate analyses confirmed that former smokers who quit within the last two years have more hospital visits than never smokers. We also found that both former smokers and current smokers have more outpatient visits than never smokers. The health and economic burden caused by cigarette smoking in the United States has been well documented [3,4,13,19,21,23]. Findings from our analysis show that current smokers were more likely to be hospitalized than never smokers. The analysis also showed that among those who had an outpatient visit in the past year, current smokers were more likely to have ≥4 outpatient visits than never smokers. Smoking has been found to have adverse health effects such as cancer, cardiovascular disease, and respiratory disease [1]. Therefore, current and former smokers are likely to have higher rates of hospitalization and outpatient visits than never smokers.

Among former smokers, we observed that inpatient and outpatient utilization (having been hospitalized, having had an outpatient visit, and among those who had an outpatient visit having >4 visits in the past year) was higher during the period immediately (<2 years) after quitting than other periods. Other population-based studies and studies among HMO populations also have observed that former smokers accrue higher health care costs during the period immediately after quitting which dissipates within a period of 1–4 years [10,13,24]. This phenomenon may be due to the fact that people who recently quit may have experienced major health events that have prompted them to quit smoking [11,25].

This study has some limitations. Estimates of cigarette smoking were based on self-reporting and were not validated with biochemical tests. However, self-reported smoking status has validity when compared with measured serum cotinine levels and yields similar population prevalence estimates [26]. Nevertheless, there is the potential for recall bias as to when the smokers actually quit. The longer the period of time since quitting, the more likely their recollection bias may be [27]. However, a study of middle-aged adults observed that they were able to accurately recall their smoking status of 20 years earlier and those who did not accurately recall their smoking status were more likely to misclassify themselves as never smokers than former smokers (which may have biased the results in our study towards the null) [28]. Secondly, since the data were from cross-sectional yearly surveys, it was not possible to analyze respondents’ past health care utilization in order to establish causality. Thirdly, we did not examine the relationship between smoking patterns (either daily/some day or amount of cigarettes consumed) and health care utilization among current smokers. Current smokers represent a heterogeneous group and include persons who have just started to smoke, persons with established patterns of nondaily smoking and established daily smokers. Blacks, Hispanics, younger smokers, and smokers with higher levels of education are more likely to be nondaily smokers than other socio-demographic groups [29]. They are also less likely to have emphysema or cancer than daily smokers [29]. Future analyses should examine the relationships between smoking patterns and health care utilization among both current and former smokers (i.e., total duration of smoking, age of initiation, and for current smokers the amount that they smoke). Despite the limitations, our use of a nationally-representative sample in our study strengthens our understanding of the relationship between smoking and health care utilization. Our findings are consistent with the results of other studies that have found smoking to increase hospitalization and outpatient utilization.

Smoking cessation has health benefits for persons of all ages [28]. For example, our results showed that the likelihood of being hospitalized in the past year was similar for former smokers of ≥2 years and never smokers. For those who continue to smoke, increased health care utilization translates into higher medical costs—therefore making smoking cessation beneficial to reducing the health-related and economic burden on the individual and society. Each year smoking costs $96 billion in direct health care expenditures, not to mention $97 billion in productivity losses. Specifically, smoking-related diseases such as lung cancer, COPD, ischemic heart disease and cerebrovascular disease are costly [4,30]. Approximately 70% of adult smokers in the United States report that they would like to quit smoking and 44% actually attempt to quit each year [2,31]. Treatments for tobacco dependence are effective and multiple attempts at quitting and repeated interventions are often needed for individuals to completely stop smoking [21]. Therefore, smokers should be encouraged to utilize available treatments including counseling and anti-smoking medications. The public health community should assist smokers—including young adults—in their attempts to quit so that more smokers can quit before they experience adverse health problems [1,32]. Persons who quit before 35 years of age have similar life expectancy as those who have never smoked [33].

Although the number of effective cessation treatments has increased over time, most smokers are not using these methods [34]. Sixty-nine percent of smokers report being advised by their physician to quit, while 22% of smokers engage in behavioral counseling (e.g., quitlines, group therapy, *etc.*) or use effective smoking cessation medications which are associated with slightly higher cessation rates [35,36]. Quitlines, which provide free smoking cessation counseling, are now available in every state and can be accessed by calling 1-800-QUIT NOW. Comprehensive tobacco control programs including, increasing the price of cigarettes, clean indoor air laws, mass media campaigns, insurance coverage for cessation therapies, and health system changes (to remind providers to counsel for tobacco use cessation) have also been shown to be effective in increasing cessation.[4,15,37]. Cigarette smoking has decreased dramatically over the past 40 years; the decrease, however, has slowed in past few years [2]. Widespread comprehensive tobacco programs should further decrease the prevalence of smoking and associated health care utilization and costs [38].

## Figures and Tables

**Table 1.** Demographic Characteristics and Health Care Utilization by Smoking Status — NHANES, 1999–2004.

	Current Smoker	Former Smoker (years since quitting)				Never Smoker
		<2 years	2–4 years	5–9 years	≥10 years	
	% (CI)	% (CI)	% (CI)	% (CI)	% (CI)	% (CI)
Overall	24.9 (23.5–26.2)	3.3 (02.8–03.8)	2.6 (02.3–02.9)	3.2 (02.8–03.6)	15.9 (14.9–16.9)	50.2 (48.5–51.9)
Gender						
Male	54.1 (52.2–56.0)	50.8 (46.4–55.2)	53.7 (47.2–60.3)	55.5 (49.8–61.2)	57.1 (54.6–59.6)	40.8 (39.5–42.0)
Female	45.9 (44.0–47.8)	49.2 (44.8–53.6)	46.3 (39.7–52.8)	44.5 (38.8–50.2)	42.9 (40.4–45.4)	59.2 (58.0–60.5)
Race/Ethnicity						
Non-Hispanic White	72.0 (68.1–75.9)	72.8 (67.0–78.6)	75.3 (69.9–80.7)	76.8 (72.0–81.5)	83.4 (80.3–86.4)	67.5 (64.0–71.1)
NH Black	11.9 (09.6–14.3)	9.0 (06.4–11.7)	8.8 (06.1–11.5)	6.8 (04.4–09.3)	6.0 (04.5–07.4)	12.8 (10.6–15.0)
Mexican-American	6.6 (04.9–08.3)	8.7 (06.3–11.0)	9.8 (06.9–12.6)	6.7 (04.0–09.3)	4.3 (02.9–05.7)	8.4 (06.6–10.2)
Other	9.5 (06.5–12.5)	9.5 (04.8–14.3)	6.1 (02.5–09.7)	9.7 (05.8–13.6)	6.4 (04..0–08.8)	11.3 (08.6–14.0)
Age Group						
18–24	13.0 (11.5–14.6)	16.8 (12.5–21.0)	11.0 (07.3–14.8)	1.8 (00.2–03.3)	0.0 (00.0–00.1)	11.1 (09.9–12.3)
25–34	22.8 (20.7–24.9)	24.0 (18.5–29.4)	21.4 (16.5–26.3)	24.0 (18.7–29.3)	2.4 (01.6–03.2)	22.1 (20.3–23.9)
35–44	25.5 (23.4–27.7)	24.2 (18.1–30.3)	18.4 (12.9–23.8)	20.9 (15.3–26.6)	14.7 (12.5–16.9)	22.2 (20.5–23.8)
45–64	32.4 (30.5–34.3)	26.4 (20.9–31.8)	33.9 (28.3–39.5)	36.8 (32.0–41.6)	46.7 (43.8–49.7)	27.9 (26.3–29.5)
≥65	6.2 (05.5–07.0)	8.7 (06.1–11.4)	15.3 (11.2–19.3)	16.5 (13.0–20.0)	36.1 (34.2–38.0)	16.7 (15.6–17.9)
Education						
More than High School	39.1 (36.4–41.9)	52.7 (47.5–58.0)	50.2 (43.9–56.5)	52.0 (46.3–57.7)	56.0 (52.7–59.3)	59.4 (57.5–61.3)
High School Diploma/GED	32.9 (30.5–35.3)	27.2 (22.4–32.0)	28.2 (23.3–33.1)	26.9 (22.1–31.7)	25.5 (23.6–27.5)	22.6 (21.1–24.0)
Less than High School	27.9 (25.9–30.0)	20.1 (16.1–24.1)	21.6 (17.4–25.9)	21.1 (16.2–26.0)	18.4 (16.1–20.8)	18.0 (16.5–19.5)
Poverty Level						
At or Above	73.0 (70.3–75.7)	77.1 (72.2–82.0)	80.2 (76.2–84.3)	83.9 (80.0–87.8)	85.4 (83.7–87.1)	79.8 (78.0–81.5)
Below	18.9 (16.2–21.6)	13.2 (09.1–17.3)	10.9 (08.3–13.6)	9.1 (07.0–11.2)	6.5 (05.2–07.8)	11.7 (10.5–13.0)
Unknown	8.0 (06.6–09.5)	9.7 (06.2–13.2)	8.8 (05.2–12.5)	7.0 (03.7–10.3)	8.1 (06.6–09.6)	8.5 (07.1–09.9)
Health Insurance						
No	30.1 (28.1–32.1)	17.3 (14.0–20.7)	20.3 (15.3–25.3)	12.6 (09.6–15.6)	6.3 (05.0–07.6)	15.2 (13.7–16.8)
Yes	69.9 (67.9–71.9)	82.7 (79.3–86.0)	79.7 (74.7–84.7)	87.4 (84.4–90.4)	93.7 (92.4–95.0)	84.8 (83.2–86.3)
Health Care Utilization						
Inpatient (# of times hospitalized)						
Hospitalized - No	89.3 (88.2–90.4)	80.4 (76.0–84.8)	86.6 (83.1–90.1)	88.9 (86.5–91.4)	87.1 (85.5–88.7)	90.0 (89.0–90.9)
Hospitalized -Yes	10.7 (09.6–11.8)	19.6 (15.2–24.0)	13.4 (09.9–16.9)	11.1 (08.6–13.5)	12.9 (11.3–14.5)	10.0 (09.1–11.0)
Hospitalized 1 time	77.1 (71.3–82.8)	81.4 (73.9–88.9)	74.8 (63.0–86.6)	76.1 (61.3–90.9)	69.0 (63.6–74.4)	78.9 (75.8–81.9)
Hospitalized ≥2 times	22.9 (17.2–28.7)	18.6 (11.1–26.1)	25.2 (13.4–37.0)	23.9 (09.1–38.7)	31.0 (25.6–36.4)	21.1 (18.1–24.2)
Outpatient (# of visits)						
0 Visit - No	23.3 (21.4–25.2)	12.9 (09.3–16.5)	17.9 (12.2–23.6)	13.1 (08.2–18.1)	8.3 (07.0–09.6)	16.3 (14.8–17.8)
≥1 Visit - Yes	76.7 (74.8–78.6)	87.1 (83.5–90.7)	82.1 (76.4–87.8)	86.9 (81.9–91.8)	91.7 (90.4–93.0)	83.7 (82.2–85.2)
1–3 Visits	58.0 (55.7–60.3)	49.4 (43.4–55.4)	48.0 (40.7–55.2)	52.2 (45.8–58.7)	50.6 (47.8–53.5)	57.9 (56.5–59.4)
≥4 Visits	42.0 (39.7–44.3)	50.6 (44.6–56.6)	52.0 (44.8–59.3)	47.8 (41.3–54.2)	49.4 (46.5–52.2)	42.1 (40.6–43.5)

**Table 2.** The Relationship Between Smoking and Health Care Utilization - NHANES, 1999–2004.

	In-Patient Services		Out-Patient Visits	
	Hospitalization (Y/N)	Number of Times Hospitalized	Visit (Y/N)	Number of Out-patient Visits
	Yes	≥2 Times	Yes	≥4
	Odds Ratio^1^ (95% CI)	Odds Ratio (95% CI)	Odds Ratio (95% CI)	Odds Ratio (95% CI)
Smoking Status				
Never	1.00 (ref)	1.00 (ref)	1.00 (ref)	1.00 (ref)
Current	1.20* (1.06–1.37)	1.13 (0.78–1.64)	0.94 (0.81–1.13)	1.18* (1.06–1.33)
Former <2 yrs	2.49* (1.86–3.34)	0.85 (0.48–1.49)	1.75* (1.15–2.65)	1.65* (1.29–2.12)
Former 2–4 yrs	1.39 (0.98–1.97)	1.19 (0.60–2.38)	1.15 (0.76–1.75)	1.59* (1.17–2.18)
Former 5–9 yrs	1.17 (0.86–1.59)	1.14 (0.50–2.57)	1.47 (0.90–2.40)	1.34* (1.03–1.74)
Former ≥10 yrs	1.22* (1.02–1.46)	1.33 (0.95–1.86)	1.75* (1.42–2.14)	1.17* (1.04–1.32)
Gender				
Male	1.00 (ref)	1.00 (ref)	1.00 (ref)	1.00 (ref)
Female	1.62* (1.47–1.80)	0.93 (0.75–1.16)	2.90* (2.56–3.30)	1.66* (1.49–1.85)
Race/Ethnicity				
NH White	1.00 (ref)	1.00 (ref)	1.00 (ref)	1.00 (ref)
NH Black	1.12 (0.96–1.32)	1.25 (0.98–1.61)	1.12 (0.96–1.32)	1.02 (0.91–1.16)
Mexican-American	0.93 (0.76–1.14)	0.95 (0.64–1.43)	0.72* (0.62–0.85)	0.89* (0.79–1.00)
Other	0.86 (0.67–1.10)	0.83 (0.48–1.41)	0.71* (0.55–0.92)	0.90 (0.74–1.10)
Age Group				
18–24	1.00 (ref)	1.00 (ref)	1.00 (ref)	1.00 (ref)
25–34	1.11 (0.82–1.49)	0.60 (0.27–1.34)	0.89 (0.71–1.11)	1.05 (0.88–1.27)
35–44	0.99 (0.77–1.26)	0.88 (0.45–1.74)	0.92 (0.74–1.15)	1.15 (0.92–1.43)
45–64	1.19 (0.94–1.51)	1.43 (0.75–2.72)	1.26* (1.02–1.56)	1.42* (1.19–1.69)
≥65	1.92* (1.54–2.39)	1.84 (0.98–3.46)	2.77* (2.16–3.55)	2.51* (2.08–3.02)
Education				
More than High School	1.00 (ref)	1.00 (ref)	1.00 (ref)	1.00 (ref)
High School Diploma/GED	1.21* (1.04–1.42)	0.81 (0.52–1.27)	0.88 (0.74–1.06)	1.10 (0.98–1.24)
Less than High School	1.63* (1.32–2.01)	1.24 (0.89–1.71)	0.75* (0.62–0.92)	1.21* (1.03–1.41)
Poverty Level				
At or Above	1.00 (ref)	1.00 (ref)	1.00 (ref)	1.00 (ref)
Below	1.59* (1.36–1.87)	1.50* (1.05–2.13)	1.05 (0.87–1.28)	1.51* (1.30–1.75)
Unknown	1.20* (1.01–1.42)	0.90 (0.57–1.41)	0.81* (0.69–0.96)	0.91 (0.77–1.08)
Health Insurance				
No - Health Insurance	1.00 (ref)	1.00 (ref)	1.00 (ref)	1.00 (ref)
Yes - Health Insurance	1.60* (1.29–2.00)	0.95 (0.61–1.49)	3.15* (2.67–3.73)	1.97* (1.68–2.32)

^1^ Adjusted Odds ratio

^2^ * p<0.05
